# Relationships among COVID-19 Prevention Practices, Risk Perception and Individual Characteristics: A Temporal Analysis

**DOI:** 10.3390/ijerph182010901

**Published:** 2021-10-17

**Authors:** Lu Wang, Jie Yu, Dongmei Chen, Lixia Yang

**Affiliations:** 1 Department of Geography and Environmental Studies, Ryerson University, Toronto, ON M5B 2K3, Canada; 2 Department of Geography and Planning, Queen’s University, Kingston, ON K7L 3N6, Canada; chendm@queensu.ca; 3 Department of Psychology, Ryerson University, Toronto, ON M5B 2K3, Canada; lixiay@ryerson.ca

**Keywords:** time, prevention behavior, risk perception, COVID-19

## Abstract

The effectiveness of public health measures in containing an infectious disease largely depends on how the general public is taking the prevention practices in daily lives. Previous studies have shown that different risk perceptions and sociodemographic characteristics may lead to vastly different prevention behaviors. This paper applies a temporal perspective in examining the changing patterns of prevention practices over time and their dynamic relationships with the perceived risk towards COVID-19 and its individual characteristics. Three key timelines (February, April, and June of 2020) were identified to represent the early, lockdown, and reopening stages of the first wave. Data were drawn from an online survey conducted in the Greater Toronto Area (GTA) of Canada (*n* = 470). Chi-square tests and logistic regression models revealed important temporal patterns in practicing different hygienic and mobility-related prevention measures and the respondents’ risk perceptions during the three timelines. The factors predicting the level of prevention practices vary across the three timelines, based on the specific type of prevention, and within the changing public health contexts. This study contributes to the literature on COVID-19 by incorporating a temporal perspective in conceptualizing prevention predictors. It provides crucial insights for developing timely public health strategies to improve infectious disease prevention at different stages and for individuals with varying backgrounds.

## 1. Introduction

Reacting to an infectious disease outbreak in the absence of a vaccination, traditional public health measures (e.g., personal hygienic practices, isolation, quarantine, social distancing, travel restriction, etc.) have been rigorously implemented to contain the spread of the disease [1,2,3]. The rapidly evolving coronavirus disease 2019 (COVID-19) pandemic has triggered numerous debates over the effective and appropriate prevention measures, policies, and public health messages among authorities and the general public, especially during the first wave of the pandemic. How the general public adheres the voluntary and mandatory prevention practices has a profound impact on the transmission and prevention of an infectious disease like COVID-19. 

Researchers from different disciplines are interested in factors shaping prevention practices [4]. A group of studies focused on the role of individual perception of the disease and risk in shaping prevention behavior to an infectious disease [5,6]. Behavioral studies have adopted different conceptual frameworks (e.g., References [7,8,9]) to illustrate the relationship between different types of risk perception and prevention measures of infectious diseases. Risk perception is generally defined as subjective judgments of a negative outcome associated with a risk [10], including different dimensions, such as cognitive, emotional, affective, personal, and societal risk perceptions [11,12]. Controlled for different individual variables, the results generally suggested that a high-risk perception of a disease is associated with an increased likelihood of practicing certain health behaviors [12]. In spatial and geographical sciences, it is well-recognized that human mobility is crucial in shaping disease transmission [13,14,15] To understand the factors shaping the mobility-related prevention of an infectious disease, studies have revealed that a higher risk perception is associated with reduced mobility [16,17,18]. Empirically, during previous pandemics, the high risk perception of an infectious disease is positively related with an increased chance of making necessary behavioral changes (e.g., Reference [19] on swine flu, [20] on SARS, and [21] on H1N1). Similarly, recent studies on the psycho-behavioral responses to COVID-19 in many locales showed that different risk perceptions lead to vastly different preventive practices [18,22,23,24]. 

Still, much of the conceptual frameworks on the relationship between prevention practices and risk perception are characterized by “interpretational ambiguity” ([2], p26). It is necessary to consider the temporal features of infectious diseases and their public health interventions in shaping prevention behaviors with different preconditions and individual characteristics [2]. However, the temporal aspect of prevention behavior and risk perception has been largely understudied [2,25] First, infectious disease prevalence carries strong spatiotemporal features that have direct impacts on local public health intervention strategies [26,27,28,29] and potentially affect individual prevention towards infectious disease [14]. The ongoing COVID-19 pandemic has highlighted the importance of bringing in a temporal perspective, since public health measures in many countries have evolved rapidly during the first wave of the pandemic. The boundaries between mandatory, recommended, and voluntary prevention practices are constantly shifting in the changing public health contexts across timelines. For example, mask wearing raised controversies during the early stage but became a mandate in a later stage when more scientific evidence became available [12]. Recent studies suggest that voluntary prevention behaviors during the early stage of the COVID-19 pandemic were critical in containing the disease, yet vastly understudied [30]. Second, a temporal analysis will help understand how individual characteristics predict prevention behaviors in relation to the risk perception over time. Most recent studies yielded mixed findings on how specific individual characteristics (e.g., age, sex, income, and education) shape prevention (e.g., References [20,30,31,32,33,34]). A limited number of studies has incorporated a temporal perspective and show that individual characteristics exert different effects on prevention behaviors over time [30], setting up a foundation for developing specific public health intervention strategies [35]. Therefore, it is crucial to situate different types of prevention practices across timelines in different places during the COVID-19 pandemic. 

This paper aims to understand the complex relationships among prevention behaviors, risk perception towards COVID-19, and individual characteristics from a temporal perspective, based on an online survey conducted in the Greater Toronto Area (GTA) of Canada. It seeks to address the following research questions: (1) How did individual prevention behaviors and risk perceptions change over time during the first wave of COVID-19? (2) How do different prevention practices relate to risk perception and individual characteristics across the timelines? The GTA is the most populous metropolitan area and the largest COVID-19 hotspot in Canada. Three key timelines during the first wave of COVID-19 in 2020 were selected to capture individual psycho-behavioral changes over time and the factors associated with such changes. They are February (early stage: very low case numbers in Canada, and public health guidelines have not been officially announced), April (lockdown stage: growing case numbers and rapid community spread, and provincial emergency orders in place), and June (reopening stage: decreasing case numbers, and restrictions were gradually relaxed). The results of the study provided important insights and timely evidence for the further analysis and modeling of infectious disease, as well as developing and implementing public health interventions in a timely manner. 

## 2. Data and Methods

The data used for this study were collected from an online survey conducted from June to November in 2020. The survey was administered among adult residents in the GTA using Qualtrics. It is part of a larger research project examining various aspects of the community response to COVID-19 in Toronto. All aspects of the survey and data collection received ethical approval from the Ryerson University Research Ethics Board (REB) and Queen’s University General Research Ethics Board (GREB). The online survey was deemed appropriate, as a traditional paper-based survey was impossible to conduct during a pandemic. Additionally, a timely and efficient survey administration through an online survey platform would help to collect more accurate responses regarding participants’ recent experiences. Recruitment was primarily done via key informants, community collaborators, and online advertisements on social media and in local newspapers and newsletters. To accommodate those interested individuals with limited Internet access, assistance was provided through the telephone to help someone to complete the survey on their behalf. A nonrandom and purposeful sampling strategy was adopted primary via referral from our community collaborators and connections. Recruiting residents of the Greater Toronto Area (GTA), we aim for balanced representations of women and men, urban and suburban residents, different age groups, etc. Due to the challenges in conducting an online survey during the pandemic, sampling bias is acknowledged as a limitation. 

In total, over 770 responses were collected, in which 300 invalid cases were removed for reasons such as an abnormally large number of autogenerated survey completions within a short time period (e.g., we have received more than 100 responses within 3 to 4 h, with answers out of contexts), those from outside of the study area, and those that did not complete the questions with key variables. In the end, 470 valid cases were included in the final sample. 

The survey included questions on prevention behaviors, the perceived risk associated with COVID-19, and the socioeconomic and demographic backgrounds of the survey respondents. The survey incorporated a temporal perspective in collecting information on prevention practices and risk perception across the three key timelines (February, April, and June). The survey was launched in June, and participants were asked to recall their experiences in February, April, and June respectively. There were two broad types of prevention practices examined: hygienic-related (i.e., hand washing, mask wearing, and sanitizing objects before entering home) and mobility-related prevention practices (i.e., cancelling social gatherings and reducing the in-person grocery shopping frequency). Questions on prevention and risk perceptions were repeated for the months of February, April, and June, and the remaining questions, such as those on socioeconomic background, did not have such a temporal dimension. 

Table 1 displays the main variables extracted from the survey and used in the study. Five prevention behaviors were treated as dependent variables for the logistic regressions that will be explained later. The risk perception and individual characteristics are explanatory variables. The four prevention variables (hand washing, mask wearing, sanitizing objects before entering home, and cancelling social gatherings) were coded as a dichotomous (“always” vs. “not always” practice) variable. Reducing the in-person grocery shopping frequency was also measured dichotomously (reduced vs. not reduced frequency). For the explanatory variables, risk perception (low, medium, or high); attitude towards public health interventions (conservative, neutral, or not conservative); age (<35, 35–54, or 55+); and household income (<$49,999 as low, $50,000–$119,999 as medium, and >$120,000 as high) were measured as three-level categorical variables; gender was a dummy (female and male) variable. The attitude towards public health interventions was measured through the views toward the timing of the first provincial lockdown in March and provincial reopening in June (e.g., those who held a conservative attitude regarded the timing of lockdown too late and reopening too soon). Risk perception was measured using the perceived level of fear towards COVID-19 (not fear as low, neutral as medium, and fear as high). This is due to the consideration that an evolving infectious disease has a strong immediate effect on risk perception compared to other disease or health conditions [21], and such immediacy is an emotional dimension to perceived risk [36]. Among the explanatory variables, risk perception was a temporal variable measuring the degree of fear towards COVID-19 for each specific timeline, while the others remained constant across different timelines.

The overall temporal changes in prevention practices and risk perceptions within the sample were tabulated to provide a general understanding of the trends and patterns. Chi-square tests were then conducted to explore the bivariate relationships between prevention and risk perception and the individual characteristics, which were all measured categorically. The initial chi-square tests on individual characteristics and prevention practices included a wider range of variables (i.e., age, gender, household income, education level, employment status, household size, having seniors or children in the household, and attitudes towards public health interventions). Only those that showed significance in at least one prevention practice at one timeline were further included in the logistic regression models. 

Separate logistic regressions were conducted for the three timelines in order to examine the temporal differences in the effects of the explanatory variables on the dependent variable (prevention practices). For each timeline, five logistic regressions were conducted for the five prevention variables, respectively. The different regression models provided critical insights into the dynamic relationships of prevention behaviors, risk perceptions, and individual characteristics. The final explanatory variables included risk perception towards COVID-19, age, household income, gender, and attitude towards public health interventions. The multicollinearity was manually tested among selected explanatory variables on their bivariate relationships, and no noticeable degree of correlation among the explanatory variables was found. 

## 3. Findings 

### 3.1. Sample Profile

Table 1 reports the main sample characteristics, as well as overall temporal changes in the hygienic- and mobility-related prevention behaviors and risk perceptions toward COVID-19 across the three timelines. Overall, Table 1 reveals high rates of compliance with different prevention practices in April and June. Except for mask wearing, the percentages of people who always practiced the other prevention measures increased greatly from February and peaked in April before declining slightly in June (reopening). This temporal pattern is seen in the percentages of those who reported a high level of risk perception. In the case of mask wearing, a consistent increase was observed from February to June, as wearing a mask in public places as a prevention measure was controversial in the early stage of the pandemic in Canada, and it was only first recommended by Canada’s chief public health officer in April and then became partially mandatory in June. 

### 3.2. Bivariate Relationship between Prevention Behavior, Risk Perception, and Individual Characteristics

Table 2 reports the results of cross-tabulation and the chi-square test of the relationships between prevention and the explanatory variables at different timelines. Risk perception has a temporally varying relation with various prevention practices. In February, proportionally, more people with a high risk perception “always” engaged in the four prevention measures (i.e., hand washing, mask wearing, sanitizing objects, and cancelling social gatherings), compared to those with low and medium risk perception levels (*p* < 0.001). In April and June, some of these associations continued to be significant, such as in the case of hand washing and mask wearing (See Table 2). 

In terms of individual characteristics, overall, being female, in the middle and older age groups, having a lower income, and having a conservative or neutral attitude towards public health intervention are associated with “always” practicing different preventions. Yet, the specific relationships vary across different timelines (see Table 2). In February, those who had lower household incomes, 35–55 years old, and female had significantly higher percentages in always practicing one or more of the prevention measures. During the lockdown stage (Apr.), some of these significant relationships continued with being female and being 35–55 years old. In addition, being 55+ and holding a conservative or neutral attitude towards public health intervention were found to be associated significantly with always cancelling social gatherings and reducing in-person grocery shopping. In the reopening stage (June), being 35–55, 55+, and holding a conservative or neutral attitude towards public health intervention exerted significant bivariate relationships with the prevention behaviors, such as cancelling social gatherings and sanitizing objects (see Table 2). 

### 3.3. Logistic Regression on Different Prevention Practices 

Table 3, Table 4 and Table 5 report the odds ratio and confidence intervals of the logistic regression models for the three timelines, respectively. Overall, the risk perception demonstrated a significant relationship in all five prevention practices in February (early stage). Such a relationship became statistically insignificant in April (lockdown stage), when age and attitude towards public health interventions started to exert stronger effects in predicting prevention behaviors. The different levels of prevention between the younger (19–34) and older age groups (55+) became more significant in June (reopening stage). Despite the general patterns, much complexity exists in how specific factors shape different types of prevention practices. 

In February (Table 3), risk perception had the strongest effect on both hygienic and mobility-related prevention practices. Those with a high risk perception were 3.67, 3.98, 2.88, and 2.86 times more likely to “always” practice all prevention measures, except reducing in-person grocery shopping, compared to those with a low risk perception. Women were 2.26 and 2.14 timely more likely than men to “always” cancel social gatherings and sanitize objects. Having a lower household income increased the likelihood of “always” wearing a mask and sanitizing objects by 147.6% and 157%, respectively, compared to their high-income counterparts. Being in the age group 35–54 significantly increased the odds by 32.5% in reducing in-person grocery shopping frequencies compared to those aged 55 years and above.

In April (Table 4), only two predictors were found significant (i.e., age and attitude towards public health intervention). Hand washing and mask wearing had no significant predictors identified. Being 35–55 years old was 1.20 times more likely to “always” sanitize objects than their older counterparts. The youngest age group was much less likely to “always” cancel social gatherings. The attitude towards public health interventions is significant in predicting mobility-related prevention practices, as those who held a conservative or neutral view towards public health interventions were more likely to “always” cancel social gatherings and reduce in-person grocery frequency than their nonconservative counterparts. 

In June (Table 5), having a conservative attitude towards public health interventions, having a high level of perceived risk, and being in the older age group were significant predictors of “always” practicing one or more prevention practices. Specifically, those who were conservative towards the public health interventions were 6.93 times more likely to “always” cancel social gatherings than their nonconservative counterparts (3.71 times for hand washing). The youngest age group was much less likely to “always” wash hands, cancel social gatherings, and sanitize objects. People who had a high risk perception were 5.93 times more likely to “always” wear a mask than those with a low perceived risk. 

## 4. Discussions

This paper addressed the research questions by revealing important temporal patterns in individual prevention behaviors and risk perceptions towards COVID-19 during the first wave of the pandemic. Overall, participants’ levels of risk perception and prevention practices aligned with the evolving COVID-19 conditions and public health measures at different timelines in the GTA. The percentage of respondents who kept a high level of prevention was high in our study. However, the factors shaping individual prevention practices varied temporally based on the specific types of prevention practices, individual risk perceptions, and characteristics within the changing public health contexts.

First, the COVID-19 prevention practices and risk perceptions at an individual level followed a clear temporal pattern across the three key timelines. Previous studies indicate that a high level of risk perception is often associated with a high level of prevention behaviors in infectious disease [5,6,7,9]. Our findings conform to the literature by revealing a similar relationship particularly evident in the early stage of the pandemic (Feb.), where there was a lack of clear public health guidelines and effective interventions in place. In April (lockdown stage) and June (reopening stage), risk perception was no longer a significant predictor of different prevention practices, except for mask wearing in June. These findings point to the importance of considering the temporal features of individual perception and public health contexts as an infectious disease evolves [2] when conceptualizing the relationship of prevention and risk perception, especially in a global pandemic such as COVID-19.

Second, our findings indicated the necessity to distinguish among different types of prevention practices and their specific temporal dimensions when analyzing respective predictors. The variation and fluidity of each prevention practice were especially evident during the COVID-19 pandemic when different public health guidelines were introduced at different points in time as the scientific knowledge of COVID-19 grew over time. Our statistical models showed distinct results in the changing predictors of different prevention practices when situated at the three timelines. For example, as a voluntary prevention measure in February, cancelling social gatherings only became a mandatory public health recommendation in April before it was allowed again with loosening social distancing measures in June. The regression models showed that the odds of “always” cancelling social gathering increased significantly in February for those who had a higher risk perception and were female and in April and June for those in the oldest age group and holding a conservative view of the public health interventions. In the case of mask wearing, respondents with a higher risk perception were more likely to wear a mask during the early and reopening stages. No single predictor was significant for the lockdown period. In Ontario, mask wearing was not a mandate in public places until after the beginning of the provincial reopening. This finding is consistent with other studies indicating that practicing mask wearing was associated with personal risk perception before it became a mandate [37]. Further, scientific knowledge on the effectiveness of different prevention practices progressed over time during the pandemic. Grounding evidence pointed to the effectiveness of mask wearing [38], while sanitizing objects was found to be less effective [39]. Our study revealed a consistent increase among the respondents who always practiced mask wearing, while a decrease in those sanitizing objects after the province reopened with different predictors. These findings suggest the complexity in understanding prevention behaviors and the necessity to analyze different types of prevention measures individually from a temporal perspective. 

Third, it is important to note the varying relationships between prevention and individual sociodemographic characteristics, such as age, income, and gender. Consistent with other studies (e.g., References [30,40]), being in the older age group significantly increased the odds of “always” practicing some prevention practices compared to their younger counterparts. Yet, the role of age in predicting prevention practices was only significant in April and June compared to February. This could be related to the growing epidemiological evidence on the greater health risks of COVID-19 for older people [41]. Our survey also revealed that those from the middle age group (35–55) tended to take prevention behaviors starting at an earlier stage of the pandemic. This is likely related to the working status and caretaking roles of the middle-aged group in households. In terms of income, our finding showed a temporal pattern. While in the early stage, the lower-income group was more likely to always practice prevention than their higher-income counterparts, and the predictability of income disappeared in April and June. This finding differs from some recent studies of COVID-19 that suggested an association between low-income status and the lower likelihood of practicing preventions [31,42]. This discrepancy could be explained by the survey sample, where 83% of the participants in the low-income group had a postsecondary education or above (i.e., university students, recent university graduates, and young professionals were part of the low-income group). Studies have shown an association between higher levels of education and higher levels of prevention [31,43,44]. Another possible explanation is that low-income individuals were more likely to use public transit for commuting for work, especially in the early stage of the pandemic [45]. Further research is needed to distinguish the variations within the low-income group and its intersections with the other individual and contextual factors. Gender is a significant factor only in the early stage of the pandemic in predicting cancelling social gatherings and sanitizing objects before entering home. This is consistent with the findings in recent studies that women tend to take pro-prevention behaviors for COVID-19 [31]. In April and June, gender is replaced by age to be a more dominant sociodemographic determinant in explaining different prevention patterns. 

Finally, this study indicates that public health interventions played a powerful role in shaping the practices of individual disease prevention. From February to April, the percentages of participants who always practiced various prevention measures increased fairly rapidly. Further, about 36% of the survey participants held a conservative attitude towards public health interventions. This variable was found significant in April and June in explaining several prevention practices. Before public health intervention started, risk perception was an important determinant of prevention practices instead. Prevention practices that were clearly recommended in the local public health guidelines at different timelines (i.e., hand washing, cancelling social gatherings, and mask wearing) were practiced more often by participants compared to self-imposed measures (i.e., sanitizing objects before entering home and reducing in-person grocery shopping). Further, the percentage of those always wearing a mask increased consistently from February to June, reflecting the effect of revised public health recommendations at that time. 

## 5. Conclusions

This paper provides crucial insights for developing timely public health strategies to improve prevention practices for individuals with different backgrounds. Recent studies of COVID-19 suggest that early-stage preventions, either self-imposed or government-imposed, are crucial in containing transmission, before a surge in cases overwhelms the tracing and testing capacities [39,46]. This paper showed in the early stage of the COVID-19 pandemic, despite a lack of effective interventions and clear public health messaging on prevention measures such as mask wearing, a considerable proportion of participants were already engaged in self-imposed prevention. Logistic regressions further indicate that such prevention behavior was largely attributed to individuals’ perceived risk towards COVID-19 in the early stage. Since the start of public health intervention, respondents holding a conservative view towards intervention were more likely to continue to practice prevention over time. These findings provided crucial evidence in rethinking the responses of public health to COVID-19 and the timing of introducing different guidelines and prevention measures. Currently, in Ontario, the indicators of adjusting public health measures are assessed based on the provincial epidemiology, health system capacity, and public health system capacity during the previous two weeks. Future response frameworks to a global pandemic like COVID-19 should consider earlier messaging to the public for timely prevention. Further, targeted public health intervention strategies should consider the local demography and temporal characteristics. Nonintrusive measures such as risk communication strategies are found to be particularly effective in targeting specific demographics and fostering better compliance [39]. Such nonintrusive measures could be useful in the early stage of the pandemic for achieving a better preparedness at the public health, community, and individual levels. 

## 6. Limitations and Future Research

A number of limitations exist in this study. Firstly, conducting a questionnaire survey during a global pandemic has demonstrated many unique challenges in terms of sampling, sample size, recruitment, and representativeness [47]. For example, the online survey tended to exclude those who did not have access to technology and was more appealing to people with higher education levels [47,48]; we also received a large number of autogenerated invalid responses for the purpose of receiving incentives. To address this limitation, assistance via telephone was provided to survey respondents with limited access to the Internet or Internet-based devices. We also conducted manual detecting for fraudsters in online research [49]. Nevertheless, these challenges led to an inevitably biased sample. There were more participants who were female (68.4%), middle-aged (45.8%), and with post-secondary education (90%) participating in the online survey. People with lower education levels were underrepresented in our sample. Secondly, risk perception is a highly complex concept that involves various cognitive and emotional dimensions [12,21]. Due to the scope of the paper and the main focus of a temporal analysis, it is hard to be inclusive of all dimensions of risk perception. Considering the strong immediate effect on risk perception of infectious disease [21], such immediacy is considered an emotional risk perception [36], and this paper used one emotional measurement of risk perception (i.e., level of fear). Finally, this study was situated in a highly populated urban center, focusing on the three temporal periods during the first wave of COVID-19. The findings should be interpreted carefully when examining other study areas and for later pandemic waves. 

The limitations lead to a number of paths for future research. Firstly, qualitative research methods can be a valuable addition to the study by identifying personal contexts and reasons for the changing psycho-behavioral responses towards COVID-19 at different stages of the pandemic. Future primary data collection through interviews and/or focus groups will be particularly helpful in providing in-depth insights to improve our understanding of the findings from the statistical analyses; reveal more nuanced patterns in the temporal changes of prevention and its predictors; and contextualize them in the wider socioeconomic, cultural, and regulatory frameworks. Secondly, future research could be conducted to expand the scope of the survey to improve the sample size and representativeness, which would also allow for incorporating a more comprehensive set of variables. For example, different types of risk perceptions (e.g., using the CoWoRP scale of probability, severity, worry, and unsafeness developed by Reference [11] to measure the risk perception) and contextual factors (neighborhood characteristics, housing, disease hotspots, etc.) could be examined using similar statistical methods. Thirdly, future research can adopt the survey and methodological framework across multiple waves and during the entire duration of the pandemic. Such a longitudinal approach would provide critical insights in establishing theoretical models and evaluating public health interventions over time. Fourthly, the nature of the online survey conducted during the pandemic undoubtedly presented many challenges for the elderly to participate in the survey. Using 55+ as the older age cut-off, along with 18–34 and 35–54, allowed for a more balanced distribution of the sample across different age groups and enabled us to compare different age groups using statistical methods. Future works can focus more specifically on older adults by expanding the data collection to explore their experiences in practicing and managing preventions. Finally, mobility-related preventions, such as reducing their daily spatial activities, is particularly effective in containing disease transmission; yet, it has complex patterns. Future work is needed to develop suitable spatiotemporal models in order to delineate the spatial activity patterns during the pandemic and identify the associated factors using a combination of datasets, such as survey, census data, and mobile phone data. A neighbourhood approach would be potentially useful in situating mobility-related prevention and spatial movement in urban neighbourhoods.

## Figures and Tables

**Table 1:** Sample characteristics and study variables: count and valid percentage.

Study Variables	*n* (%)		
Explanatory variables			
**Gender**			
Female	321 (69.0)		
Male	144 (31.0)		
**Age**			
18–34	171 (36.5)		
35–54	215 (45.8)		
55 and over	83 (17.7)		
**Household Income (before tax)**			
$30,000-$49,999	178 (39.3)		
$50,000-$119,999	172 (38.0)		
$120,000 and over	103 (22.7)		
**Attitude towards public health intervention**			
Conservative	171 (43.3)		
Neutral	147 (37.2)		
Not conservative	77 (19.5)		
**Risk Perception**	**Feb.**	**Apr.**	**June**
High	126 (31.6)	202 (50.6)	170 (42.6)
Medium	135 (33.8)	97 (24.3)	156 (39.1)
Low	138 (34.6)	100 (25.1)	73 (18.3)
Dependent Variables	**Feb.**	**Apr.**	**June**
**Wash Hands** (Always)	295 (73.0)	387 (95.8)	383 (94.8)
**Wear a Mask** (Always)	160 (39.9)	348 (86.8)	366 (91.3)
**Sanitize Objects** (Always)	137 (34.1)	277 (69.1)	256 (63.8)
**Cancel Social gathering** (Always)	167 (41.5)	362 (90.3)	320 (80.0)
**In-person Grocery Shopping** (Reduced)	198 (46.2)	348 (81.3)	321 (75.2)

Note. Feb. and Apr. are the abbreviations for the study timelines February and April. Variables are bolded.

**Table 2:** Cross-tabulation between prevention practices and individual characteristics across three timelines: percentage ^1^ and chi-square value ^2^.

	Feb. (Early Stage)					Apr. (Lockdown Stage)					June (Reopening Stage)				
	Wash Hands	Wear a Mask	Sanitize Objects	Cancel Social Gathering	In-Person Grocery Shopping	Wash Hands	Wear a Mask	Sanitize Objects	Cancel Social Gathering	In-Person Grocery Shopping	Wash Hands	Wear a Mask	Sanitize Objects	Cancel Social Gathering	In-Person Grocery Shopping
**Risk Perception ^3^**															
Low	59.9	22.8	20.4	25.0	38.0	95	84.8	69.0	89.9	82.3	89.0	81.9	61.6	76.4	70.0
Medium	74.8	43.7	38.5	49.6	52.3	91.7	79.2	66.7	90.6	77.7	94.8	89.6	60.8	76.6	80.8
High	84.7	51.6	42.6	48.0	45.2	98.0	90.9	70.1	89.9	83.6	97.0	96.4	67.1	83.7	73.5
Chi-square	25.3 ***	38.0 ***	29.1 ***	30.3 ***	5.6	12.9 *	9.1	7.0	1.1	1.5	8.7	13.7 **	1.5	3.0	3.8
**Gender ^4^**															
Female	75.5	42.1	37.1	46.9	46.4	97.4	86.3	70.5	91.5	83.4	95.3	90.4	65.7	81.5	76.2
Male	66.9	33.9	26	29.4	46.9	92.1	87.4	65.4	87.3	77.5	93.7	92.9	59.1	76.2	73.6
Chi-square	3.3	2.4	4.8 *	10.9 **	0.0	6.0 *	0.1	1.1	1.8	2.0	0.4	0.7	1.6	1.5	0.3
**Household Income ^4^**															
Low	78.8	49.4	45.5	47.1	45.6	96.8	87	71.2	90.3	79.4	93.6	92.2	64.3	78.7	74.4
Medium	68.5	37	28.1	37.7	50.9	96.6	89	69.9	90.4	84.2	96.6	92.5	66.4	79.5	77.2
High	70.0	28.9	26.7	38.9	41.7	93.3	82.2	62.2	88.8	80.2	94.4	87.8	58.4	81.8	75
Chi-square	4.6	10.8 *	13.3 **	3.1	2.2	2.0	2.3	2.3	0.2	1.3	1.4	1.8	1.6	0.3	0.4
**Age ^4^**															
<35	68.0	40.8	26.5	39.5	45.5	93.2	86.4	54.4	84.2	76.8	92.5	90.5	46.9	67.1	73.4
35–55	79.1	42.0	39.2	47.0	49.8	97.8	89.0	80.7	92.8	86.5	95.1	91.2	76.1	87.2	77.5
55 +	68.0	32.9	36.5	32.4	37.8	96.0	82.2	69.9	95.9	76.7	98.7	93.2	67.6	87.8	72.6
Chi-square	6.3 *	1.9	6.0 *	5.0	3.1	4.3	2.1	26.2 ***	10.1 **	6.7 *	3.9	0.4	30.4 ***	23.8 ***	1.1
**Attitude Towards Public Health Intervention ^4^**															
Conservative	74.9	43.3	35.7	43.9	47.1	96.5	90.6	71.9	94.1	83.3	97.1	94.7	65.5	85.5	78.1
Neutral	70.5	34.9	31.5	42.5	46.9	97.3	87	67.1	91.8	86	95.9	89.0	61.6	80.1	78.2
Not conservative	77.9	42.7	34.2	35.5	38.4	93.5	82.7	68.0	82.9	72.6	90.9	92.0	67.1	71.1	67.1
Chi-square	1.6	2.5	0.6	1.6	1.8	2.0	3.2	1.0	8.3 *	6.2 *	4.7	3.5	0.8	7.4 *	3.9

* *p* < 0.05, ** *p* < 0.01, and *** *p* < 0.001. ^1^ Refers to the percentages of those who “always” practiced a prevention measure (e.g., always washed hands) and those who reduced in-person grocery shopping. ^2^ Results are shown as a percentage (%) within each subgroup. ^3^ Risk perception is a temporal variable measured in individual timelines. ^4^ These are nontemporal variables.

**Table 3:** Logistic regression analysis assessing the factors associated with each prevention practice in February (early stage): odds ratio and 95% confidence interval.

	Wash Hands	Wear a Mask	Sanitize Objects	Cancel Social Gatherings	Reduce In-Person Grocery Shopping
**Risk Perception**					
High	3.67 (1.97–7.06) ***	3.98 (2.23–7.09) ***	2.86 (1.57–5.19) **	2.88 (1.64–5.06) ***	1.13 (0.67–1.91)
Medium	2.21 (1.26–3.85) **	2.87 (1.63–5.04) ***	2.50 (1.40–4.47) **	3.123 (1.80–5.42) ***	1.77 (1.07–2.95) *
Low ^1^	1.0	1.0	1.0	1.0	1.0
**Age**					
Under 35	0.90 (0.46–0.74)	1.22 (0.614–2.42)	0.51 (0.26–1.02)	1.25 (0.63–2.45)	1.24 (0.66–2.34)
35–55	1.46 (0.74–2.86)	1.27 (0.65–2.49)	0.94(0.49–1.82)	1.66 (0.87–3.18)	1.33 (0.71–2.46) *
55 and over ^1^	1.0	1.0	1.0	1.0	1.0
**Household Income**					
Low	1.86 (0.95–3.62)	2.48 (1.34–4.57) **	2.57(1.38–4.78) **	1.52 (0.84–2.74)	1.13 (0.64–1.99)
Medium	0.90 (0.48–1.69)	1.34 (0.72–2.47)	1.03 (0.55–1.93)	0.91 (0.50–1.64)	1.48 (0.84–2.59)
High ^1^	1.0	1.0	1.0	1.0	1.0
**Gender**					
Female	1.51 (0.90–2.54)	1.57 (0.96–2.56)	1.64 (0.99–2.73) *	2.26 (1.38–3.70) **	0.96 (0.62–1.54)
Male ^1^	1.0	1.0	1.0	1.0	1.0
**Attitude Towards Public Health Intervention**					
Conservative	0.84(0.42–1.68)	1.05 (0.57–1.92)	1.07 (0.57–2.01)	1.52 (0.82–2.82)	1.45 (0.80–2.61)
Neutral	0.70 (0.34–1.39)	0.82 (0.43–1.52)	0.99 (0.52–1.88)	1.57 (0.84–2.94)	1.48 (0.81–2.69)
Not conservative ^1^	1.0	1.0	1.0	1.0	1.0

* *p* < 0.05, ** *p* < 0.01, and *** *p* < 0.001. ^1^ Reference group.

**Table 4:** Logistic regression analysis assessing the factors associated with the level of prevention in April (lockdown stage): odds ratio and 95% confidence interval.

	Wash Hands	Wear a Mask	Sanitize Objects	Cancel Social Gatherings	Reduce In-Person Grocery Shopping
**Risk Perception**					
High	2.84 (0.53–15.05)	1.95 (0.91–4.19)	1.23 (0.65–2.32)	0.89 (0.34–2.31)	1.16 (0.57–2.39)
Medium	0.46 (0.11–1.95)	0.72 (0.34–1.53)	0.98 (0.52–1.87)	1.13 (0.37–3.49)	0.88 (0.39–1.96)
Low ^1^	1.0	1.0	1.0	1.0	1.0
**Age**					
Under 35	0.35 (0.07–1.85)	1.05 (0.44–2.49)	0.52 (0.27–1.01)	0.23 (0.07–0.81) *	0.73 (0.31–1.62)
35–55	1.53 (0.23–10.07)	1.60 (0.67–3.80)	2.20 (1.12–4.32) *	0.60 (0.16–2.19)	1.54 (0.68–3.51)
55 and over ^1^	1.0	1.0	1.0	1.0	1.0
**Household Income**					
Low	2.44 (0.54–10.99)	1.31 (0.58–2.97)	2.08 (1.10–3.91)	1.70 (0.69–4.22)	1.05 (0.49–2.23)
Medium	1.56 (0.35–6.96)	1.37 (0.60–3.13)	1.53 (0.83–2.83)	1.48 (0.61–3.61)	1.22 (0.57–2.63)
High ^1^	1.0	1.0	1.0	1.0	1.0
**Gender**					
Female	2.47 (0.77–7.96)	0.834 (0.41–1.68)	1.04 (0.63–1.71)	1.43 (0.70–2.90)	1.58 (0.87–2.84)
Male^1^	1.0	1.0	1.0	1.0	1.0
**Attitude Towards Public Health Intervention**					
Conservative	2.22 (0.56–8.73)	1.90 (0.84–4.29)	1.23 (0.65–2.32)	4.13 (1.65–10.35) **	1.85 (0.90–3.79)
Neutral	2.53(0.62–10.41)	1.57 (0.71–3.47)	0.98 (0.52–1.87)	2.52 (1.05–6.04) *	2.17 (1.03–4.59) *
Not conservative ^1^					

* *p* < 0.05, ** *p* < 0.01, and *** *p* < 0.001. ^1^ Reference group.

**Table 5:** Logistic regression analysis assessing factors associated with the level of prevention in June (reopening stage): odds ratio and 95% confidence interval.

	Wash Hands	Wear a Mask	Sanitize Objects	Cancel Social Gatherings	Reduce In-Person Grocery Shopping
**Risk Perception**					
High	3.87 (0.98–15.26)	5.82 (1.94–17.52) **	0.90 (0.47–1.72)	1.35 (0.61–2.98)	0.99 (0.50–1.96)
Medium	2.30 (0.65–8.13)	2.06 (0.83–5.10)	0.75 (0.39–1.42)	0.89 (0.41–1.92)	1.57 (0.76–3.23)
Low ^1^	1.0	1.0	1.0	1.0	1.0
**Age**					
Under 35	1.2E−8 (4.3E−9–3.6E−8) ***	0.43 (0.11–1.63)	0.43 (0.23–0.83) *	0.19 (0.07–0.48) **	0.65 (0.31–1.39)
35–55	1.3E−8 (1.3E−8–1.3E−8)	0.48 (0.13–1.83)	1.63 (0.85–3.13)	0.70 (0.26–1.86)	0.88 (0.42–1.86)
55 and over ^1^	1.0	1.0	1.0	1.0	1.0
**Household Income**					
Low	0.50 (0.12–2.14)	1.48 (0.53–4.13)	1.63 (0.89–2.98)	1.03 (0.47–2.24)	1.03 (0.53–2.01)
Medium	0.87 (0.20–4.01)	1.21 (0.45–3.26)	1.54 (0.85–2.80)	0.80 (0.37–1.72)	1.12 (0.57–2.15)
High ^1^	1.0	1.0	1.0	1.0	1.0
Gender					
Female	1.31 (0.45–3.83)	0.86 (0.36–2.05)	0.99 (0.61–1.59)	1.38 (0.78–2.45)	1.20 (0.71–2.04)
Male ^1^	1.0	1.0	1.0	1.0	1.0
**Attitude Towards Public Health Intervention**					
Conservative	6.93 (1.78–26.98) **	1.99 (0.65–6.08)	0.94 (0.50–1.74)	3.71 (1.49–6.35) **	1.55 (0.80–3.0)
Neutral	2.86 (0.88–9.28)	0.79 (0.28–2.20)	0.84 (0.45–1.58)	1.71 (0.85–3.42)	1.55 (0.79–3.04)
Not conservative ^1^					

* *p* < 0.05, ** *p* < 0.01, and *** *p* < 0.001. ^1^ Reference group.

## Data Availability

The data are not publicly available due to restrictions of research ethics and privacy policy ordered by Ryerson University and Queen’s University.
